# Case report: Late in-stent thrombosis in a patient with vertebrobasilar dolichoectasia after stent-assisted coil embolization due to the discontinuation of antiplatelet therapy

**DOI:** 10.3389/fneur.2023.1129816

**Published:** 2023-04-12

**Authors:** Zhe Ji, Wanxin Yang, Yongjie Ma, Lisong Bian, Guilin Li, Yongjuan Fu, Yueshan Piao, Hongqi Zhang

**Affiliations:** ^1^Department of Neurosurgery, Xuanwu Hospital, Capital Medical University, Beijing, China; ^2^Department of Neurosurgery, Beijing Haidian Hospital, Peking University, Haidian, China; ^3^Department of Pathology, Xuanwu Hospital, Capital Medical University, Beijing, China

**Keywords:** aneurysm—dissecting, stent assisted coil embolization, thrombosis, case report, autopsy

## Abstract

Vertebrobasilar dolichoectasia (VBD) is a rare type of cerebrovascular disorder with high rates of morbidity and mortality. Due to the distinct pathological characteristics that fragmented internal elastic lamina and multiple dissections, VBD is difficult to treat and cured. Stent-assisted coil embolization is one of the main treatment modalities for such lesions. However, the duration of healing remained questionable, and there were no effective measures for evaluating endothelial coverage. Before complete endothelial coverage, the discontinuation of antiplatelet therapy may lead to fatal in-stent thrombosis; however, continued antiplatelet therapy could also result in bleeding complications. Thus, we present an autopsy case of late in-stent thrombosis due to the discontinuation of antiplatelet therapy and systematically review the literature to provide a reference for endovascular treatment and antiplatelet regimen of VBD.

## Introduction

Vertebrobasilar dolichoectasia (VBD) is a rare type of cerebrovascular disorder resulting in ectasia, elongation, and tortuosity of the vertebrobasilar artery. However, there are no current data on the exact incidence of VBD in the general population. Flemming et al. (1) assumed that the incidence was <0.05%, while Ince et al. (2) revealed that VBD was detected in ~2.06% of the first-ever stroke population. Patientswith VBD commonly present with ischemic stroke, intracranial hemorrhage, and compression of the brainstem and/or cranial nerves (3–9), which could lead to high rates of morbidity and mortality (10). Among endovascular treatments, stent-assisted coil embolization is one of the main treatment modalities for VBD.

In-stent thrombosis is one of the most common complications after stent-assisted coil embolization, the rate of which is ~1.5% (1). Postoperative antiplatelet therapy is routinely administered to prevent the occurrence of in-stent thrombosis. However, the duration of post-procedure antiplatelet therapy remains questionable. Some studies provide recommendations for the optimal duration according to clinical experience, but there is no definite evidence of timing for the discontinuation of antiplatelet therapy (2, 3). Thus, we present an autopsy case of late in-stent thrombosis due to the discontinuation of antiplatelet therapy and systematically review the literature.

## Case report

**Figure 1:**
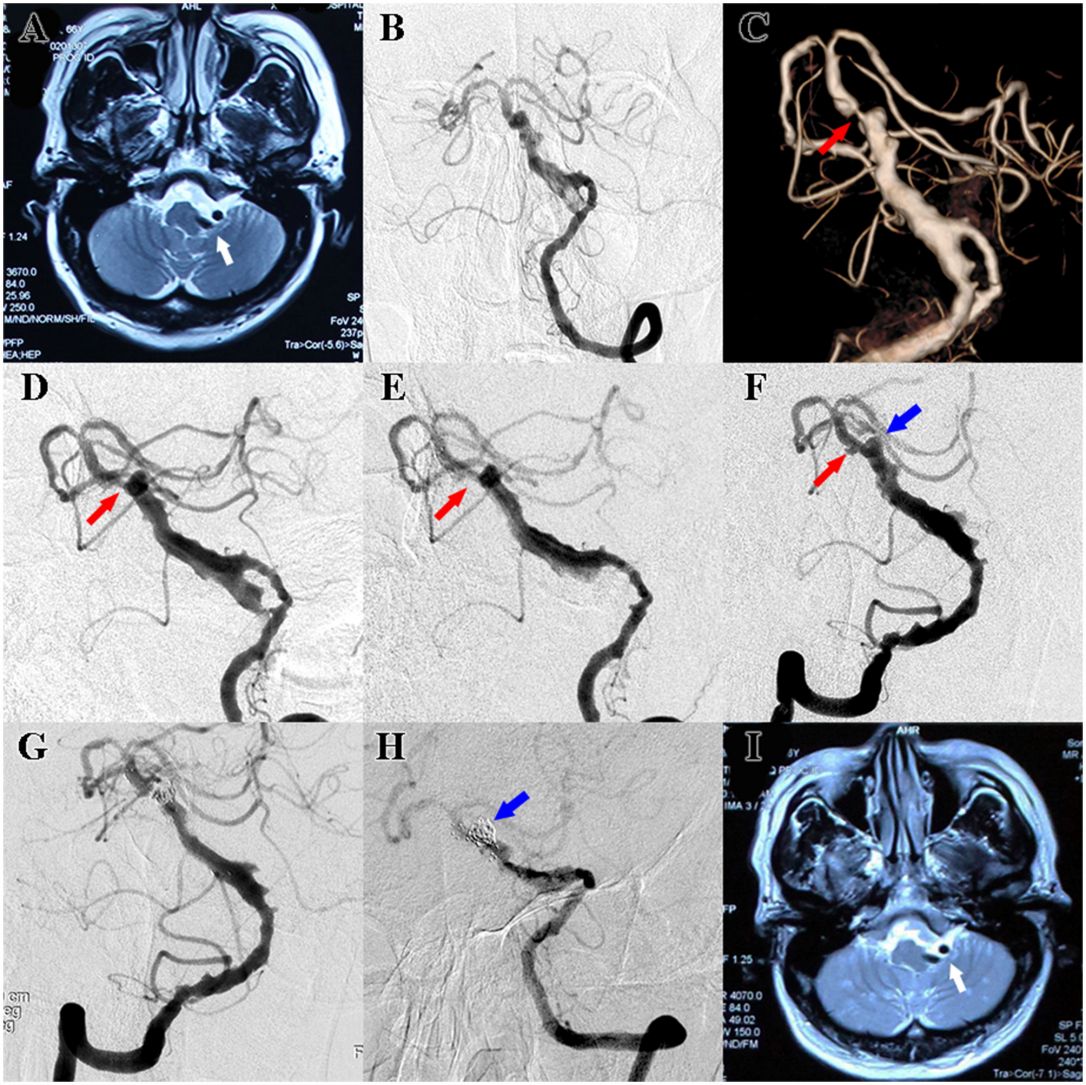
**(A)** Preoperative T2-weighted MRI showed facial nerve compression resulting from VBD (white arrow). **(B)** Standard frontal view of preoperative DSA also revealed ectasia, elongation, and tortuosity of the vertebrobasilar artery, and the diagnosis of VBD was confirmed. **(C)** 3D volume rending reconstructed image of preoperative DSA showed severe stenosis of the basilar artery (red arrow). **(D)** 2D angiography of optimal view of preoperative DSA demonstrated stenosis of the basilar artery, but the degree of stenosis was less severe (red arrow). **(E)** Postoperative angiography after balloon angioplasty along with stent implantation showed that the stenosis was partly improved (red arrow). **(F)** 6-month follow-up angiography showed that the stenosis of the basilar was improved (red arrow) while the aneurysm seemed enlarged (blue arrow). **(G)** Postoperative angiography after stent-assisted coils embolization showed that the aneurysm was occluded (blue arrow). **(H)** Follow-up angiography after stent-assisted coils embolization showed that an aneurysm-like protrusion of the VBD was occluded (blue arrow). **(I)** Follow-up MRI indicated facial nerve compression remained (white arrow).

A 66-year-old man presented with hemifacial spasm on the left side and hemiplegic paralysis on the right side, as well as lower extremity weakness. CTA revealed ectasia, elongation, and tortuosity of the vertebrobasilar artery, which indicated VBD. T2-weighted MRI showed that the aberrant vertebrobasilar artery contributed to facial nerve compression, which corresponded to the clinical symptoms (Figure 1A). Digital subtraction angiography (DSA) was also performed, and the diagnosis of VBD was confirmed. Meanwhile, severe stenosis of the basilar artery was detected according to the 3D volume rending reconstructed image. 2D angiography of optimal view demonstrated the stenosis, but the degree of stenosis was less severe. Balloon angioplasty along with stent implantation was performed to treat the stenosis. Postoperative angiography showed that the stenosis was partly improved (Figures 1B–E). Daily dual antiplatelet therapy (DAPT) including aspirin 100 mg and clopidogrel 75 mg was administrated postoperatively since then. Follow-up angiography was conducted 6 months later, which showed that the stenosis was improved while the existence of aneurysm remained. Stent-assisted coil embolization was performed to reduce the bleeding risk (Figures 1F, G). At 15 months after the endovascular treatment, follow-up angiography was performed and showed that an aneurysm-like protrusion of the VBD was occluded, but MRI indicated that facial nerve compression remained (Figures 1H, I). Furthermore, the patient complained of suffering from hemifacial spasms and desired further treatment. The surgical plan of microvascular decompression was then formulated. Because near-complete occlusion was observed in the recent angiography while DAPT was administrated continuously for ~2 years, DAPT was discontinued for 1 week before the operation to avoid the risk of perioperative bleeding without alternative short-term anticoagulation therapy. Adhesions between the tortious vertebral artery and the facial nerve were confirmed and dissected, and the operation was uneventful. Unfortunately, the patient presented with unconsciousness 8 h post-operation. A mobile bedside computed tomography (CT) scan was immediately performed and revealed cerebral infarction, while cerebrovascular ultrasound indicated the occlusion of the basilar artery, which highly indicated in-stent thrombosis.

**Figure 2:**
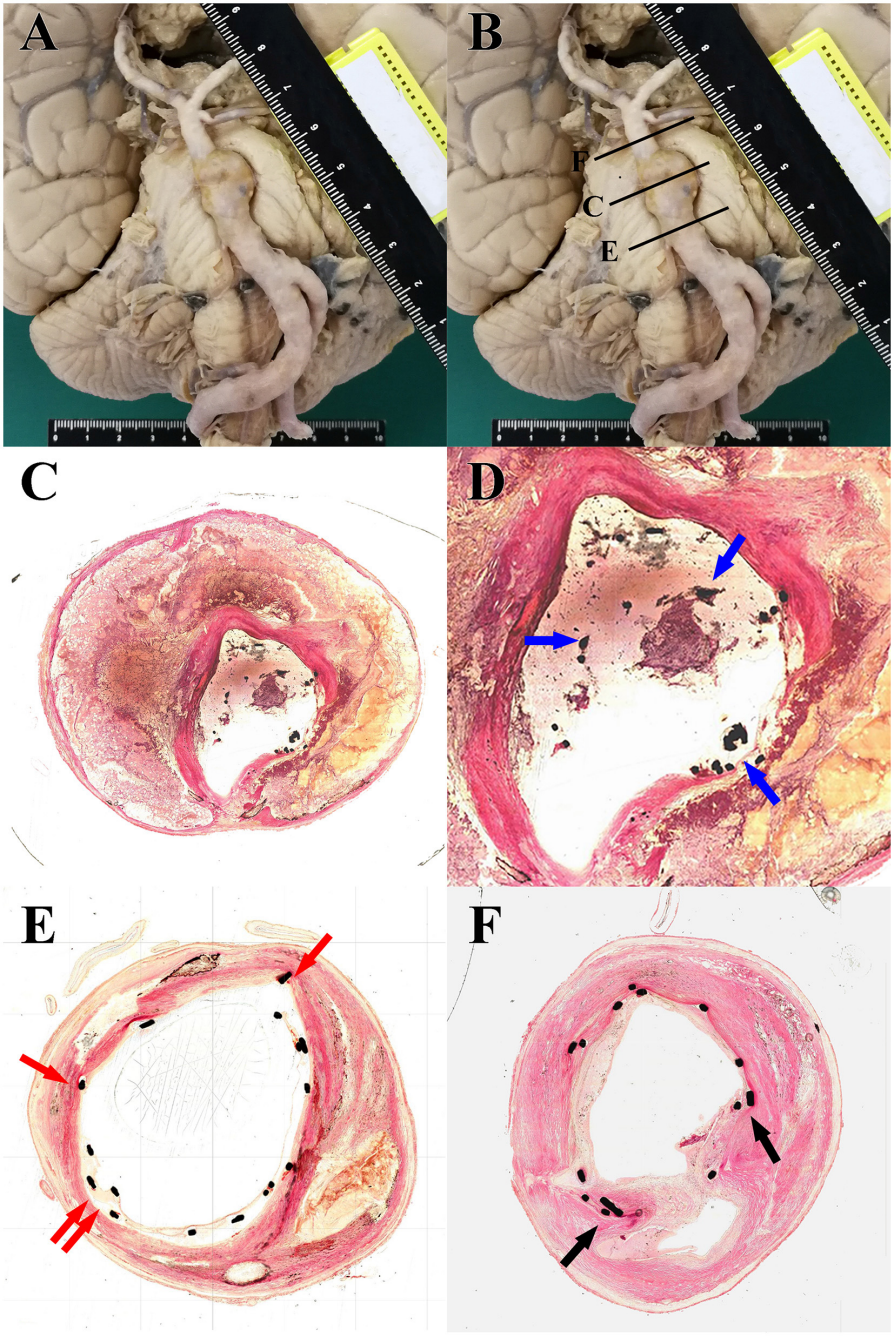
**(A)** Gross specimens of the vertebrobasilar system showed ectasia, elongation, and tortuosity of the vertebrobasilar artery accompanied by brain stem compression caused by VBD. **(B)** Schematic diagram of tissue slice position: the proximal region (E), distal region (F), and apparent dilatated region (C). **(C)** Light microscopy images from H–E staining (20 ×) of tissue slice of the apparent dilatated region: irregular intravascular lumen, loss of smooth muscle, and multiple thromboses among the dissection. **(D)** Locally magnified images (40 ×) focusing on the intravascular lumen of the section of the apparent dilatated region showed that the stent was suspended, and there was no vascular endothelium coverage on the surface (blue arrow). **(E)** Light microscopy images from H-E staining (40 ×) of tissue slice of the proximal region: the stent was relatively adherent, and vascular endothelium or connective tissue coverage appeared above the stent (red arrow). **(F)** Light microscopy images from H–E staining (40 ×) of tissue slice of the distal region: smooth muscle coverage could be observed above the stent (black arrow).

An autopsy was performed with the agreement of relatives. Gross specimens of the vertebrobasilar system showed VBD with obvious protrusion located at the middle of the basilar artery, where stent-assisted coil embolization was performed. Brain stem compression caused by VBD could also be observed (Figure 2A). The specimen of the vertebrobasilar system was dissected separately, which was then chemically fixed and embedded in resin. Three specified regions of VBD, the proximal region, the distal region, and the apparent dilatated region were cut into sections, and microscopic observation was performed (Figure 2B). The section of the apparent dilatated region showed that the stent was suspended, and there was no vascular endothelium coverage on the surface (Figures 2C, D), which was evident and likely contributed to the in-stent thrombosis after the discontinuation of antiplatelet therapy. For the proximal region, the stent was relatively adherent, and vascular endothelium or connective tissue coverage appeared above the stent (Figure 2E). For the distal region, even, smooth muscle coverage could be observed on the surface of the stent (Figure 2F).

## Discussion

In contrast to saccular aneurysms and regular dissecting aneurysms, VBD presents a fragmented internal elastic lamina combined with multiple dissections in pathology (4). The distinct pathological characteristics may account for why VBD is difficult to treat and takes longer to cure. In the past, conventional stent-assisted coil embolization or conventional stent implantation was used to treat VBD, but the efficacy was limited. LEO stents were proven to be an effective endovascular treatment modality in a previous study, which showed that most patients had good reconstruction of the target vessels and improvement of symptoms shortly after treatment (5). However, the results with long-term follow-up suggested that the clinical outcome was poor despite a good radiological outcome (6). Patients who presented with compressive symptoms had an even worse prognosis after endovascular treatment, which may not be beneficial for such patients (7). Recently, a flow diverter was used to treat VBD. Nevertheless, a systematic review and meta-analysis suggested that there were no statistically significant differences in favorable clinical outcomes, complete/near-complete occlusion, or complications between stent-assisted coiling and flow diverter groups in treating posterior circulation non-saccular aneurysms, which are similar to VBD (8). Furthermore, the morbidity and mortality rates of VBD patients who receive surgical treatment are also extremely high (9). Overall, the optimal treatment modality for VBD remains controversial.

In-stent thrombosis is one of the most common complications after stent-assisted coil embolization. A systematic review and meta-analysis revealed that ischemic/thromboembolic events and in-stent thrombosis were the most common complications, and the rate of in-stent thrombosis was ~1.5% (1). Postoperative antiplatelet therapy is routinely administered to prevent the occurrence of in-stent thrombosis. However, the duration of post-procedure antiplatelet therapy remains questionable. Early discontinuation of antiplatelet therapy may result in ischemic complications, while continuous antiplatelet therapy could increase the risk of hemorrhagic complications. For conventional stents, one retrospective study including 395 patients with 403 aneurysms treated with stent-assisted coil placement concluded that DAPT for more than 9 months and late switching to monotherapy are recommended for its prevention (2). Kim et al. suggested that longer-term DAPT (>9 months) should be considered after stent-assisted coil embolization for unruptured intracranial aneurysms, although its efficacy remains to be clarified (10). Noah Hong et al. (3) suggested that the optimal time to discontinue might be ~18–36 months after stent-assisted coil embolization. Large cohort-based studies or randomized clinical trials are warranted to confirm these results. However, the earlier three studies had limitations because none of them provided data on the rates of major bleeding for patients treated with antiplatelet therapy. A prospective randomized multicenter trial was conducted to compare the effect of short-term (6 months) and long-term (12 months) DAPT on UIAs in patients undergoing stent-assisted coil embolization to find the optimal duration, and the results were highly anticipated (11). For flow diverters, a systematic review and pooled analysis indicated that a duration of post-procedure clopidogrel therapy <6 months was associated with greater rates of ischemic complications than a clopidogrel regimen of ≥6 months for pipeline embolization of cerebral aneurysms, while the duration of postprocedural high-dose ASA therapy was also not associated with ischemic complications (12). Another meta-analysis indicated that clopidogrel therapy ≤6 months is associated with higher rates of thrombotic events, but high-dose ASA >6 months is associated with fewer permanent thrombotic and hemorrhagic events (13). DAPT had been continuously administered for ~2 years for the case presented, which was much longer than the so-called long-term DAPT in previous studies. However, the fatal basilar thrombo-ischemic event still occurred. All the studies mentioned earlier only provide recommendations for the optimal duration based on clinical experience, but there is no definite evidence or guidelines for the timing of DAPT discontinuation.

None of the routine imaging examinations, such as CT, MR, or DSA, could evaluate the situation of endothelial coverage above the stent, so the evidence provided by these imaging examinations for the timing of DAPT discontinuation is limited. Currently, another imaging method is being used to evaluate cerebral vascular disease: optical coherence tomography (OCT). OCT images can show more features at the pathological level, especially stent apposition and neointima formation (14). A study of coronary stenting demonstrated that incomplete apposition led to a delay in neointimal coverage of the stent struts, activation of platelet function, and late stent thrombosis (15). The study by Rouchaud et al. (16) demonstrated that good wall apposition was a key factor for aneurysm occlusion after flow diverter treatment in a histological evaluation of rabbits. Guerrero et al. (17) described the assessment of the endothelial healing of recurrent intracranial aneurysms after treatment with a PED shield at 8 weeks post-implantation by using OCT. These findings corresponded to our pathological results, which provided valuable support for the perspective that the better the stent adherence is, the easier it is to be covered by the intima or even the muscular layer. When complete endothelial coverage was confirmed in OCT, antiplatelet therapy could be confidently discontinued, whereas for those patients with incomplete intimal coverage, longer or even lifelong medication may be necessary (18). Nevertheless, there were still some limitations to using OCT in the neurovascular field. Stiff catheters and tortuous intracranial arteries may result in iatrogenic dissection and poor imaging quality (19, 20).

## Conclusion

This case indicated that the discontinuation of long-term antiplatelet therapy for VBD patients treated with stent-assisted coil embolization could be a cause of late in-stent thrombosis and subsequent cerebral infarction, which may result in poor clinical outcomes. In regard to patients with VBD, the safety of discontinuation of long-term DAPT after endovascular treatment should be carefully considered.

## Data availability statement

The original contributions presented in the study are included in the article/supplementary material, further inquiries can be directed to the corresponding author.

## Ethics statement

Written informed consent was obtained from the individual's legal guardian/next of kin for the publication of any potentially identifiable images or data included in this article.

## Author contributions

ZJ, WY, and YM drafted and revised the manuscript and were involved in the acquisition of data. ZJ and WY contributed equally to the manuscript. LB was involved in the acquisition of data. YF and YP performed the autopsy. GL and HZ supervised and revised the manuscript. All authors contributed to the article and approved the submitted version.
